# Interrater reliability of the BelRAI Social Supplement in Flanders, Belgium: Simultaneous rating of community-dwelling adults with care needs during COVID-19

**DOI:** 10.3389/fpsyg.2022.941648

**Published:** 2022-09-16

**Authors:** Shauni Van Doren, David De Coninck, Kirsten Hermans, Anja Declercq

**Affiliations:** ^1^LUCAS—Center for Care Research and Consultancy, KU Leuven, Leuven, Belgium; ^2^Centre for Sociological Research, KU Leuven, Leuven, Belgium

**Keywords:** Belgium, BelRAI Social Supplement, home care services, interRAI, interrater reliability, instrument evaluation, needs assessment, social context

## Abstract

**Background:**

The BelRAI Screener is a short-form assessment consolidating internationally validated interRAI items focusing on physical and psychological aspects of functioning and problems with activities of daily living. It was fully implemented in the Flemish home care setting as of June 2021. In a biopsychosocial model for developing a personalized and effective care plan social and contextual aspects are considered equally important to biomedical ones. Thus, a social supplement to the BelRAI Screener was collaboratively developed with stakeholders and tested to gather additional information on the social context of community-dwelling adults with care needs.

**Objective and methods:**

To examine the interrater reliability of the BelRAI Social Supplement in Flanders, Belgium, an observational study was conducted using a convenience sample. The method of simultaneous rating was used due to strict COVID-19 guidelines at the time and to minimize assessment burden. Fifty two community-dwelling adults requesting home care support were simultaneously assessed by two independent assessors during home visits. Interrater reliability was tested on all 80 items of the BelRAI Social Supplement using observed agreement, kappa coefficients, and intraclass correlation coefficients.

**Results:**

The kappa mean (0.74) and median (0.79) values for nominal items, show substantial agreement, while the kappa mean and median values for ordinal items were 0.81 and 0.90, which correspond to almost perfect agreement. Following the traditional cut-off points for the interpretation of the kappa statistic, reliability was almost perfect (κ > 0.81) for 49% of all items, substantial (0.60 < κ ≤ 0.80) for 33%, moderate (0.40 < κ ≤ 0.60) for 8%, and poor (κ < 0.40) for 10%. The majority of items with poor kappa value, showed a high observed agreement, reflecting homogeneity of the sample rather than poor agreement.

**Conclusion:**

The strength of kappa agreement for the items in this version of the BelRAI Social Supplement is generally substantial to almost perfect, with high proportions of observed agreement. COVID-19 restrictions had a large impact on the planning and execution of the home visits. A final optimization of the instrument and accompanying manual according to the findings will result in an improved version ready for nation-wide implementation.

## Introduction

The aging of populations is a long-term trend which significantly strains the capacity and performance of regional and national health and social systems. This growing share of the elderly population is associated with an increase in demand for health and social care services, including the use of social care at home (Tarricone and Tsouros, 2008; Hood et al., 2022). Along with an increase in the number of older persons with care needs staying in their communities, we see a rise in the concurrence of comorbidities and in complexity of care needs (Fried et al., 2004; Valderas et al., 2009). These converging trends result in a pressing need for an update of the (mostly fragmented) health care systems, in which care providers, interventions and policies focus on one aspect of a person’s complex care needs (North, 2020). The time and resources constraints due to this fragmentation obstruct the process of assessing the complexity of a person’s health-related and contextual characteristics (Whiteneck et al., 2004; North, 2020).

In 2016, the World Health Organization (WHO) developed a framework on integrated people-centered health services that highlights a holistic and multi-disciplinary outpatient approach to caregiving (World Health Organization [WHO], 2015). In this framework, a person with care needs is at the center of a network that consists of their family and close community. This people-centered approach acknowledges the close bond between the individual and their social and cultural capital, with persons and their communities considered to be active participants in health and social care services. The health services within this framework strive toward providing holistic care (i.e., treating not just the disease, but the whole person), using an out-patient approach (i.e., treatment does not require hospitalization), and is accessible across different levels of health care delivery (Kraus de Camargo, 2011; van Dulmen et al., 2015).

A standardized way of holistically assessing a person with disabilities—and sharing those findings with other care professionals—is an important step toward translating and implementing the WHO-model in practice (Berg et al., 2009; Van Doren et al., 2021a). Comprehensive care assessments, such as the interRAI suite of instruments, provide professionals with a standardized way to evaluate a person’s functional status and social context characteristics (Heckman and Jónsson, 2018).

The interRAI suite of instruments is internationally validated and contains a variety of comprehensive assessment tools developed with a particular setting or population in mind (home care, long-term care facilities, mental health care, palliative care, etc.) (Gray et al., 2009). Contrary to other disease- or population-specific assessment tools, all interRAI instruments share a set of core items and terminology. By providing a universal language to the people working in different health and social services settings, the transfer of information between health professionals, administrators, and governments improves. This facilitates multi- and interdisciplinary assessments, providing person-oriented care and making informed decisions at the policy-level (Carpenter and Hirdes, 2013; Hirdes et al., 2020).

In 2018, the Belgian government opted for a mandated implementation of the interRAI instruments in all care sectors in order to translate the WHO’s framework in practice (Vandeurzen, 2018; World Health Organization [WHO], 2019). This process created a demand for the cultural adaptation and the translations of the interRAI instruments into Dutch, French and German, the three national languages of Belgium. The collection of these translated instruments is called BelRAI (Declercq et al., 2009).

Since June 2021, the first BelRAI instrument—the BelRAI Screener–was fully implemented in the home care setting in Flanders (i.e., the Dutch-speaking region of Belgium) (Vermeire, 2020). The BelRAI Screener is a short-form assessment consolidating internationally validated interRAI items focusing on physical and different aspects in the biopsychosocial model of disability (Engel, 1980; Borrell-Carrió et al., 2004). This Screener allows professionals to obtain a rapid and standardized first assessment of a person’s care needs and gauge whether someone needed a full BelRAI Home Care assessment. The assessment also allows for the calculation of a dependency and care complexity index to checks a person’s eligibility for a regional care budget (Vermeulen et al., 2015; Van Doren et al., 2021b). As of 2023, the BelRAI Home Care instrument will be implemented in home care and the BelRAI Long-Term Care Facilities instrument will be implemented in nursing homes.

Although the BelRAI Screener is positively evaluated by professionals, especially those active in social care mention the lack of socially oriented items (Vermeulen et al., 2015; Vernimmen et al., 2018). The context a person lives in has an impact on their functioning as well. To answer this question, a BelRAI Social Supplement was developed. This Social Supplement is implemented in the whole of Flanders as of June 2022. While the collaborative development and extensive testing of the BelRAI Social Supplement previously were described (Van Doren et al., 2021a,^2022^), testing the interrater reliability of the instrument had not been done yet.

Evaluating the reliability of the assessment and quality of the data is important for all users of the assessments and their output (Wellens et al., 2012a,b; Van Doren et al., 2021b). One way of evaluating an instrument is by analyzing the consistency in coding between two raters assessing the same person in a short time span. Low interrater reliability endangers the validity of the assessment, as it is important that the results are reproducible and consistent between two assessors. In the past, there have been a handful of international studies evaluating the interrater reliability of several interRAI instruments (Martin et al., 2007; Gray et al., 2008; Hirdes et al., 2008; Wellens et al., 2012c; Kim et al., 2015; Boscart et al., 2021). Based on these results, the respective instruments, manuals and training materials and their translations have been updated and improved. The purpose of this study is thus to test the interrater reliability of the BelRAI Social Supplement among persons requesting home care in Flanders, Belgium. This study was a collaborative effort with the Flemish Government and partners in the field.

## Materials and methods

An observational study was conducted using a convenience sample of persons seeking home care support in Flanders, the northern region of Belgium. Community-dwelling adults with care needs (physical or mental) or disabilities are included. Furthermore, the person had to be able to speak the native language (Dutch) and give its consent for an assessment. Persons applying for maternity care or other short-term care and support were excluded. To determine interrater reliability, two independent assessors completed the BelRAI Social Supplement for the same person with a care need.

Gwet (2014) provides an overview of the minimum number of assessments needed to achieve a desired confidence interval for interrater reliability studies. We chose to aim for a confidence interval of 85% in this study, for which Gwet (2014) states that we would need to complete at least 44 assessments. This seemed a feasible number in the context of this study.

With regards to the recruitment of social care workers, we selected a limited number of social care workers who had been trained in completing the BelRAI Screener as well as the Social Supplement, and who had previous experience with administering both during a home visit. We created a directory of all eligible assessors so that home care services were able to contact the appropriate candidates within their own organizations. Interested assessors were in turn asked to contact us to receive further instructions and schedule their usual home visits on the approved dates. All participating assessors (*n* = 9) needed to attend an (online) refreshment course of about 3 h in total with information about completing the BelRAI Social Supplement and the practical arrangements.

One trained social care worker and one of the researchers (SVD)^1^ simultaneously completed the BelRAI Social Supplement during a home visit according to standard interRAI conventions. The evaluation was based on a semi-structured interview and observations made during the conversation. Both assessors were blinded to the others’ results and were not permitted to discuss the case with each other, nor to exchange information after the home visit.

During the home visit, the social care worker (rater one) guided the home visit to provide the appropriate services and to complete the BelRAI Screener and the BelRAI Social Supplement. The second rater (SV) shadowed the social worker and simultaneously completed the BelRAI Social Supplement based on the conversation and their own observations. The second rater remained in the background during the assessment.

We reported this study in accordance with the Guidelines for Reporting Reliability and Agreement Studies (GRRAS) to make interpretation and synthesis of other reliability studies possible (Kottner et al., 2011).

### Ethical approval and consent to participate

We obtained ethics approval to conduct this study from the Social and Societal Ethics Committee of KU Leuven (file number G- 2019 05 1654). All potential participants were provided with full information about the study during the scheduling process of the home visit. During field testing, all persons receiving or requesting social care services, or their representatives were required to complete a written informed consent agreement before the start of the assessment. Refusal did not affect the care provided to the person.

### BelRAI Social Supplement

The BelRAI Social Supplement is a supplement to the BelRAI Screener and other interRAI instruments that gathers information on the social context of community-dwelling adults with care needs. The instrument and its manual have known several iterations as this study is part of the final stage of a larger evidence-based policy research project using the revised Medical Research Council (MRC) framework for the design, evaluation and implementation of complex interventions (Craig et al., 2013; Van Doren et al., 2022). The version of the BelRAI Social Supplement that was used in this study has skip patterns^2^ and consists of a maximum of 80 items that are categorized into four sections [for more information on these sections, see Van Doren et al. (2021a)]:

A. Environmental assessment;
B. Civic engagement;
C. Psychosocial well-being; and
D. Informal care and support.

### Statistical analysis

We compared individual items from the BelRAI Social Supplement between the two assessments using the proportion of observed agreement and kappa coefficients. Proportion of observed agreement (Po) is the ratio of exact agreement between the assessors as a function of the total number of assessments. The Kappa coefficient is a measure that represents the agreement between two assessors, corrected for chance. For nominal items, unweighted Cohen’s kappa (κ) was used. For ordinal items, weighted quadratic Cohen’s kappa (κw) was used. Kappa coefficient values—and thus, the strength of the association—were considered to be poor when below 0.40, moderate when ranging between 0.41 and 0.60, substantial from 0.61 to 0.80, and almost perfect from 0.81, in line with Landis and Koch (1977).

For dummy variables or binary items, bias and prevalence effects may result in skewed agreement parameters (Byrt et al., 1993; Hoehler, 2000; Vach, 2005). As highlighted by Wellens et al. (2012c), the prevalence of item scores affects the stability of the kappa coefficients. If the assessments of the sample of individuals are homogeneous (and thus, lack variability), it is unlikely that κ will approximate the maximum score of 1. This is independent of sample size. To tackle this, we calculated the prevalence index (Sim and Wright, 2005): the absolute value of the difference between the number of cases rated as positive by both raters, and the number of cases rated as negative by both raters; divided by the total number of assessments. Bias represents the degree to which the assessors disagree on the share of cases in a category. It affects the interpretation of the magnitude of κ. When bias is sizeable, kappa tends to be higher than when bias is low or absent (Feinstein and Cicchetti, 1990; Byrt et al., 1993). It is calculated by taking the absolute value of the difference between the number of cases rated as positive by rater A and negative by rater B, and the number of cases rated as negative by rater A and positive by rater B; divided by the total number of assessments.

In addition to the weighted kappa coefficient, intraclass correlation coefficients (ICC, two-way random model, absolute agreement) and 95% confidence intervals (95% CI) were calculated for ordinal items (Fleiss and Cohen, 1973; Laschinger, 1992; Field, 2005). All tests were conducted in SPSS Version 26.

## Results

Between August and November 2021, nine assessors participated and a total of 52 individuals with care needs at home were included in the study. The assessors were trained social workers active at Flemish social care services organizations.

The mean age of participating adults with care needs was 72 years (range: 35–92), and 71% was female. The majority of our sample (67.3%) was over 65. 36.5% of the sample owned their place of living with no current loan or mortgage, 34.6% rented from a social housing association, and 19.2% rented on the private housing market. The remaining 9.7% either owned with a current mortgage or loan or had an alternate living arrangement (e.g., cohabitating with family members for free).

Demographic information about the individuals with care needs and the assessors can be found in Table 1.

**TABLE 1:** Demographic information on community dwelling adults with care needs (*n* = 52) and assessors (*n* = 9).

	Statistic (mean or %)

Community-dwelling adults with care needs (*n* = 52)	
**Age (average)**	72.1 years
(Less than) 50 years old	5.8
51–60 years old	13.5
61–70 years old	21.2
71–80 years old	25.0
81–90 years old	30.8
Older than 90 years old	3.8
**Gender**	
Male	28.8
Female	71.2
**Living situation**	
Owner, with loan or mortgage	7.7
Owner, without loan or mortgage	36.5
Renter, social housing company	34.6
Renter, private market	19.2
Other	1.9

**Assessors (*n* = 9)**	

**Gender**	
Male	11.1
Female	88.9
**Organization**	
Family care and complimentary home care services	57.7
Social work services	42.3

The agreement statistics in Table 2 show that the level of agreement between assessors was generally high. When calculating the kappa mean (0.74) and median (0.79) values for nominal items, we observed substantial agreement. The kappa mean and median values for ordinal items were 0.81 and 0.90, which both correspond to almost perfect agreement. Following the traditional cut-off points for the interpretation of the kappa statistic, reliability was almost perfect (κ > 0.81) for 49% of all items, substantial (0.60 < κ ≤ 0.80) for 33%, moderate (0.40 < κ ≤ 0.60) for 8%, and poor (κ < 0.40) for 10%.

**TABLE 2:** Agreement statistics for each item of the BelRAI Social Supplement (*n* = 52).

Item (# categories)	P_o_	*K*	CI of κ	Strength agreement	Prevalence index	Bias index	ICC	CI of ICC

Section A: Environmental assessment								
Number of persons in the home (4)	0.98	0.97*	0.90–1.00	Almost perfect			0.97	0.94–0.98
Number of children (5)	0.96	0.93*	0.79–1.00	Almost perfect			0.95	0.92–0.97
Living conditions (6)	0.90	0.85	0.71–0.98	Almost perfect				
Disrepair of the home (2)	0.94	0.37	0.00–0.93	Poor	0.90	0.02		
Squalid conditions (2)	0.96	0.00	0.00–0.01	Poor	0.96	0.00		
Inadequate heating or cooling (2)	0.98	0.85	0.55–1.00	Almost perfect	0.87	0.02		
(Lack of) personal safety (2)	0.94	0.74	0.45–1.00	Substantial	0.75	0.02		
Limited access to home/rooms (2)	0.73	0.20	0.00-0.47	Poor	0.58	0.04		
Presence of pets (3)	0.87	0.73	0.55–0.91	Substantial				
Home adapted to care needs (3)	0.89	0.77	0.58–0.95	Substantial				

**Section B: Civic engagement**								

Possibility to go outside when wanted (2)	0.96	0.92	0.81–1.00	Almost perfect	0.19	0.04		
Limited use of public transport (3)	0.92	0.88	0.76–1.00	Almost perfect				
Aids—Mobility (2)	0.96	0.92	0.80–1.00	Almost perfect	0.27	0.00		
Aids—Eating (2)	0.98	0.66	0.03–1.00	Substantial	0.94	0.02		
Aids—Grooming (2)	0.94	0.88	0.75–1.00	Almost perfect	0.21	0.02		
Aids—Communication (2)	0.90	0.70	0.46–0.94	Substantial	0.60	0.06		
Aids—Safety (2)	0.96	0.89	0.73–1.00	Almost perfect	0.58	0.04		
Request for extra aid (2)	0.92	0.84	0.69–0.99	Substantial	0.19	0.08		
Extra aid—Mobility (2)	0.92	0.79	0.51–1.00	Substantial	0.16	0.11		
Extra aid—Eating (2)	1.00	1.00	1.00–1.00	Almost perfect	0.79	0.00		
Extra aid—Grooming (2)	0.94	0.58	0.18–0.98	Moderate	0.53	0.16		
Extra aid—Communication (2)	0.94	0.86	0.59–1.00	Almost perfect	0.53	0.05		
Extra aid—Safety (2)	0.98	0.89	0.68–1.00	Almost perfect	0.21	0.05		
Problems with Dutch language (2)	0.83	0.56	0.32–0.80	Moderate	0.48	0.13		
Understanding Dutch (6)	0.75	0.24*	0.00–0.65	Poor			0.26	0.00–0.73
Speaking Dutch (6)	0.69	0.63*	0.17–1.00	Substantial			0.66	0.10–0.91
Reading Dutch (6)	0.67	0.68*	0.36–0.99	Substantial			0.70	0.10–0.93
Writing Dutch (6)	0.73	0.79*	0.57–1.00	Substantial			0.81	0.39–0.93
First language (14)	0.81	1.00	1.00–1.00	Almost perfect				
IT–skills: Performance (7)	0.79	0.67*	0.35–0.99	Substantial			0.68	0.38–0.85
IT–skills: Capacity (7)	0.71	0.91*	0.83–0.99	Almost perfect			0.91	0.85–0.95

**Section C: Psychosocial well-being**								

Conflict with family/Friends (5)	0.83	0.59*	0.30–0.87	Moderate			0.59	0.38–0.74
Conflict or repeated criticism of PCG (2)	1.00	1.00	1.00–1.00	Almost perfect	0.81	0.00		
Length of alone time during day (4)	0.65	0.75	0.66–0.85	Substantial				
Preference for being alone (4)	0.90	0.81	0.65–0.97	Substantial				
Degree of loneliness (5)	0.92	0.98*	0.97–1.00	Almost perfect			0.98	0.97–0.99
In last 3 days, how often did person…								
Feel little interest or pleasure in things (4)	0.77	0.61*	0.35–0.88	Substantial			0.70	0.52–0.82
Feel anxious, restless, uneasy (4)	0.90	0.98*	0.95–1.00	Almost perfect			0.98	0.96–0.99
Feel sad, depressed, hopeless (4)	0.87	0.90*	0.79–1.00	Almost perfect			0.91	0.84–0.95
Feel happy, cheerful, content (4)	0.79	0.88*	0.75–1.00	Almost perfect			0.88	0.79–0.93
Person has confidant (2)	0.96	0.89	0.73–1.00	Almost perfect	0.58	0.04		
Person has positive outlook (2)	0.98	1.00	1.00–1.00	Almost perfect	0.27	0.00		
Strong and supportive family relationship (2)	1.00	1.00	1.00–1.00	Almost perfect	0.46	0.00		
Strong and supportive friend relationship (2)	0.90	0.80	0.65–0.97	Substantial	0.13	0.02		
Sense of involvement in community (2)	0.89	0.76	0.58–0.94	Substantial	0.19	0.00		
Major life stressors last 90 days (2)	0.87	0.72	0.53–0.91	Substantial	0.21	0.02		
Participation of social activities of interest (5)	0.69	0.84*	0.72–0.96	Almost perfect			0.85	0.74–0.91
Visit with/of family or long relation (5)	0.90	0.74*	0.45–1.00	Substantial			0.75	0.59–0.85
Other interaction with family/long relation (5)	0.92	0.98*	0.96–1.00	Almost perfect			0.98	0.97–0.99
Change in social activities last 90 days (4)	0.89	0.76	0.57–0.95	Substantial				
Trade-off because of limited funds (2)	0.98	0.94	0.83–1.00	Almost perfect	0.60	0.02		
Change in behavior due to finances (2)	0.92	0.75	0.52–0.98	Substantial	0.62	0.04		
Ability to make ends meet (6)	0.89	0.96*	0.93–0.96	Almost perfect			0.97	0.94–0.98

**Section D: Informal care and support**								

Primary informal carer for other person (2)	0.92	0.73	0.49–0.98	Substantial	0.65	0.04		
Relationship to person 1 (9)	0.92	1.00	1.00–1.00	Almost perfect				
Relationship to person 2 (9)	0.92							
Hours a week spent on informal care (6)	0.92	1.00	1.00–1.00	Almost perfect				
Unable to continue caring activities (2)	0.89	0.22	0.00–0.64	Poor	0.29	0.43		
Loss of income due to caring activities (2)	0.96	0.00	0.00–0.00	Poor	0.71	0.29		
Number of informal carers (8)	0.89	0.94*	0.88–1.00	Almost perfect			0.94	0.90–0.97
Support by informal carers (2)	0.71	0.42	0.15–0.69	Moderate	0.66	0.03		
Relationship to ICG 1 (9)	0.89	0.81	0.61–1.00	Substantial				
Relationship to ICG 2 (9)	0.92	0.84	0.55–1.00	Almost perfect				
Lives with ICG 1 (3)	1.00	1.00	1.00–1.00	Almost perfect				
Lives with ICG 2 (3)	1.00	1.00	1.00–1.00	Almost perfect				
(Almost) daily contact with ICG 1 (3)	0.94	0.76	0.51–1.00	Substantial				
(Almost) daily contact with ICG 2 (3)	0.90	0.78	0.50–1.00	Substantial				
Hours a week spent on care—ICG 1 (6)	0.83	0.91*	0.80–1.00	Almost perfect			0.91	0.84–0.95
Hours a week spent on care—ICG 2 (6)	0.85	0.92*	0.86–0.99	Almost perfect			0.93	0.83–0.97
IADL help (2)	0.94	0.37	0.00–0.90	Poor	0.87	0.08		
ADL help (2)	0.90	0.73	0.52–0.95	Substantial	0.13	0.08		
Help with child care of other dependents (2)	0.96	0.47	0.00–1.00	Moderate	0.89	0.00		
Companionship (2)	0.98	0.66	0.03–1.00	Substantial	0.92	0.03		
Social inclusion (2)	0.81	0.36	0.07–0.64	Poor	0.47	0.21		
ICG available in case of emergency (2)	0.98	0.79	0.38–1.00	Substantial	0.87	0.03		
Plans for alternative living situation in future (3)	0.77	0.55	0.32–0.77	Moderate				
ICG unable to continue caring activities (2)	0.81	0.70	0.54–0.87	Substantial	0.50	0.10		
ICG have feelings of distress, anger, depression (2)	0.89	0.77	0.61–0.93	Substantial	0.15	0.05		
ICG reports loss of income (2)	0.92	0.84	0.69–1.00	Almost perfect	0.70	0.10		
ICG dissatisfied with support PCG (2)	0.89	0.79	0.62–0.96	Substantial	0.80	0.00		

Mean	0.89	0.76			0.54	0.06	0.82	
Median	0.91	0.79			0.58	0.04	0.91	
Range	0.65–1.00	0.00–1.00			0.13–0.96	0.00–0.43	0.26–0.98	

*Weighted kappa for ordinal items (κ_w_). CI, 95% confidence interval; ICC, intraclass correlation coefficient; κ, Kappa; P_o_, proportion of observed agreement; PCG, professional care giver; ICG, informal care giver.

Agreement appears to be poor for eight out of 80 items. Three of these items are related to housing conditions: assessments regarding the disrepair of the house (κ = 0.37, P_o_ = 0.94), squalid living conditions (κ = 0, P_o_ = 0.96), and limited access to the dwelling or rooms in the dwelling (κ = 0.20, P_o_ = 0.73). The other five items are about the person’s understanding of the Dutch language (κ = 0.24, P_o_ = 0.75), their ability to continue caring activities for other persons (κ = 0.22, P_o_ = 0.89), the loss of income due to caring activities (κ = 0.00, P_o_ = 0.96), the IADL help they receive (κ = 0.37, P_o_ = 0.94), and help with social inclusion (κ = 0.36, P_o_ = 0.81).

For 41 binary items, we calculated the bias and prevalence index. The bias index ranged between 0 and 0.43, the prevalence index between 0.13 and 0.96. Mean and median observed agreements were 0.89 and 0.91, respectively. For 84% of all items, the observed agreement was greater than 0.80.

## Discussion

This study is part of the final stage of a larger evidence-based policy research project about developing and testing a BelRAI Social Supplement using the revised Medical Research Council (MRC) framework for the design, evaluation and implementation of complex interventions (Craig et al., 2013; Van Doren et al., 2022). Testing the interrater reliability of the instrument is a key step prior to the final evaluation and implementation of the BelRAI Social Supplement in the field as of June 2022. In the present study, two independent raters simultaneously assessed home care clients. Evaluating the reliability of the assessment and quality of its data is important for all stakeholders who use the collected data (for care planning, policymaking and/or financing). When its users start questioning the (perceived and actual) quality of the data, this “uncertainty can, in turn, create a laissez-faire attitude and an incorrect use of the assessment and output in practice” (Wellens et al., 2012a,b; Van Doren et al., 2021b, p.3).

Individual items which comprise the BelRAI Social Supplement were examined using the Cohen’s kappa statistic. Strength of kappa agreement was in general substantial to almost perfect, with high proportions of observed agreement. This indicates that there is little disparity between raters on most items and that interrater reliability is high. Agreement appears to be poor for eight out of 80 items. Six items had a poor kappa value, despite a high observed agreement [disrepair of the home (κ = 0.37, P_o_ = 0.94), squalid conditions (κ = 0.00, P_o_ = 0.96), unable to continue caring activities (κ = 0.22, P_o_ = 0.89), loss of income due to caring activities (κ = 0.00, P_o_ = 0.96), IADL help (κ = 0.37, P_o_ = 0.94), social inclusion (κ = 0.36, P_o_ = 0.81)]. This combination reflects a lack of variety in answers on the particular items (homogeneity), which impacts the kappa value (Sim and Wright, 2005; Vach, 2005). This result can be supported by these items showing high values on the prevalence index.

However, two items showed a low kappa value (κ < 0.40) in combination with a low proportion of observed agreement (P_o_ < 0.80) [limited access to home/rooms (κ = 0.20, P_o_ = 0.73) and understanding Dutch (κ*_*w*_* = 0.24, P_o_ = 0.75)]. Despite the fact that these items initially seem easy to assess, the manual contains a large number of guidelines to correctly code both items. While the first item is a dummy, the second item uses a Likert scale with six different answer options to define a person’s ability to understand Dutch. Based on the results of the present study, we can provide suggestions for optimization of both the items and the manual, for example adding more precise guidelines for scoring and adding examples to clarify the differences between the coding categories.

Considering the frailty of the population and the strict COVID-19 guidelines in place at the time of our data-collection, we decided to use the method of simultaneous rating for all cases. Simultaneous rating has three major benefits in our study: (1) it minimizes the burden of assessment for people with care needs and their caregivers, (2) it limits the inaccuracies of people’s self-reports and rating differences due to chances in past few days (Wellens et al., 2012b), and, (3) it reduces the odds of spreading COVID-19.

In this study, we used the method of simultaneous rating instead of separate raters. This method differs from other studies on the interRAI instruments for persons living at home (Hirdes et al., 2008; Kim et al., 2015). In those studies, a social worker or registered nurse assesses the person on their regular home visit while a second assessor has to repeat the assessment within 72 h. This way, each assessor can choose how to steer the conversation and obtain all the answers to complete the instrument. However, Kim et al. (2015, p. 224) clarify that “some home care cases were assessed simultaneously by the paired independent raters because of the limited visitation schedule during the observation period for the assessment.”

### Limitations

COVID-19 restrictions had a large impact on the planning and execution of the home visits for our data collection. This study has been postponed multiple times over a period of a year and half. Initially, we planned an in-person refreshment course for all participating assessors to provide the training for the new version of the BelRAI Social Supplement. Instead, we chose to organize an online refreshment course to limit personal contact and minimize the time commitment for the social workers who were already severely under pressure because of delays in their daily activities. Nevertheless, we only selected assessors who received a full training on the previous version of the BelRAI Social Supplement, attended multiple discussion groups and used the instrument with their clients for 10 assessments during a testing phase of the larger scale project (Van Doren et al., 2022). Additionally, we planned to complete 100 home visits, but due to the unpredictable nature of COVID-19 surges, we finalized the data collection in November 2021, when Belgium entered its fourth wave of infections/hospital admissions and a contact restrictions were announced (Federal Public Service of Health, Food Chain Safety and Environment, 2022).

Due to the skip pattern used in this version of the BelRAI Social Supplement, one item in section D (Informal care and support) regarding the nature of the person’s relationship with a second informal carer (“Relationship to person 2” in Table 2) had too few observations to conduct the analysis on (*n* = 4).

## Conclusion

Based on simultaneous rating of 52 community-dwelling adults in Flanders, Belgium, we calculated the interrater reliability of the latest version of the Social Supplement to the BelRAI Screener and Home Care instrument. Based on Cohen’s kappa and intraclass correlation coefficients, we found that 82% of items had substantial to almost perfect reliability. This indicates that there is limited disparity between raters on most items and that interrater reliability is high. A small number of items had a low kappa value, which was likely to a low number of and homogeneity of responses. Overall, our findings indicate that the BelRAI Social Supplement is ready to be used in Flemish home care assessments, with the provision that some items and the corresponding manual require further fine-tuning.

## Data availability statement

The raw data supporting the conclusions of this article will be made available by the authors, without undue reservation.

## Ethics statement

The studies involving human participants were reviewed and approved by Social and Societal Ethics Committee of KU Leuven (SMEC). The patients/participants provided their written informed consent to participate in this study.

## Author contributions

SVD, KH, and AD developed the research design. SVD collected the data and analyzed them with DDC. SVD led the writing of the manuscript. All authors contributed to the interpretation of findings, reviewed multiple versions of the manuscript, and approved the submitted version.

## Footnotes

1 SVD was the main researcher in this study. She has multiple years of experience with using the different versions of the BelRAI Social Supplement.

2 Skip patterns or branching questions are used to alter the sequential flow of a survey or instrument based on the answers to prior questions, or other known information about the respondent. This allows respondents to skip questions or sections not relevant for them (IPSOS, 2016).
